# “*They don't care to study it*”: Trust, race, and health care experiences among patient‐caregiver dyads with multiple myeloma

**DOI:** 10.1002/cam4.7297

**Published:** 2024-05-21

**Authors:** Shakira J. Grant, Jiona A. Mills, Joseph Telfair, Gabriell Erisnor, Tanya M. Wildes, Lauren C. Bates‐Fraser, Andrew F. Olshan, Erin E. Kent, Hyman B. Muss, Paul Mihas

**Affiliations:** ^1^ Division of Hematology The University of North Carolina at Chapel Hill Chapel Hill North Carolina USA; ^2^ Lineberger Comprehensive Cancer Center The University of North Carolina at Chapel Hill Chapel Hill North Carolina USA; ^3^ Gillings School of Global Public Health The University of North Carolina at Chapel Hill Chapel Hill North Carolina USA; ^4^ Jiann‐Ping Hsu College of Public Health, Georgia Southern University Statesboro Georgia USA; ^5^ School of Medicine City University of New York New York New York City USA; ^6^ Division of Hematology/Oncology University of Nebraska Medical Center/Nebraska Medical Center Omaha Nebraska USA; ^7^ Department of Allied Health Sciences The University of North Carolina at Chapel Hill Chapel Hill North Carolina USA; ^8^ Division of Medical Oncology The University of North Carolina at Chapel Hill Chapel Hill North Carolina USA; ^9^ Odum Institute for Research in Social Sciences, The University of North Carolina at Chapel Hill Chapel Hill North Carolina USA

**Keywords:** access to care, caregivers, healthcare disparities, medical mistrust, multiple myeloma, qualitative research, trust

## Abstract

**Background:**

Medical mistrust, rooted in unethical research, is a barrier to cancer‐related health care for Black/African American (AA) persons. Understanding trust, mistrust, and health care experiences is crucial, especially in multiple myeloma (MM), which disproportionately burdens Black/AA persons in incidence and survival.

**Study Purpose:**

This study qualitatively examines the experiences of Black/AA and White dyads (patient with MM and adult caregiver) to gain insights into these phenomena.

**Methods:**

From November 2021 to April 2022, we recruited 21 dyads from the UNC Lineberger Comprehensive Cancer Center. Participants completed a sociodemographic survey and a 60–90 min semi‐structured interview. We used ATLAS.ti v9 for project management and to facilitate data analysis using the Sort and Sift, Think and Shift approach (ResearchTalk Inc).

**Results:**

We interviewed 21 racially concordant dyads (11 Black/AA, 10 White) with mean patient ages of 70 (Black/AA) and 72 (White) at enrollment. Both Black/AA and White caregivers had a mean enrollment age of 68. The mean duration from MM diagnosis to enrollment for all patients was 5.5 years. Four key themes emerged: (1) knowledge and trust, (2) heightened emotions and discomfort, (3) differing mental constructs of health care experiences, and (4) mitigating mistrust, which varied by self‐identified race. Black/AA participants had greater knowledge of historical events like the U.S. Public Health Service Untreated Syphilis Study at Tuskegee and carried the emotional burden longer. They also emphasized self‐learning and self‐guided research about MM for informed medical decision‐making. Both Black/AA and White dyads emphasized the pivotal role of patient‐provider relationships and effective communication in fostering trust and addressing concerns.

**Conclusion:**

Our study offers contextual insights into the enduring challenges of medical mistrust, particularly within the Black/AA community, and its implications for patients and caregivers accessing and receiving MM‐related care. Future studies should leverage these insights to guide the development of multilevel interventions addressing medical mistrust within the Black/AA community.

## Graphical abstract

This qualitative study examined the role of trust and mistrust in the health care experiences of twenty‐one Black/African American (Black/AA) and White patient‐caregiver pairs affected by multiple myeloma. Findings reveal the profound impact medical mistrust has on health care experiences especially among Black/ . This research sets the foundation for future studies to further examine and address the health‐related impacts of mistrust..

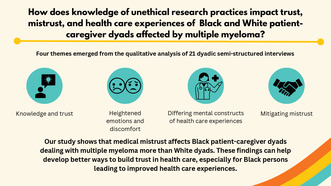

## 1 INTRODUCTION

Medical mistrust, characterized by skepticism and lack of confidence in health care providers and institutions, influences behaviors and outcomes in oncology, including cancer screenings, treatment adherence, and participation in biomedical research.^1^, ^2^, ^3^, ^4^ Disparities in cancer‐related outcomes exist among racial/ethnic groups, including those with multiple myeloma (MM).^5^, ^6^, ^7^

MM, the 14th most prevalent cancer in the United States (U.S.), with around 35,000 cases annually,^7^ demonstrates significant disparities in incidence and death rates between Black/African American (AA) and White persons.^8^ These disparities stem from variations in risk factors, disease biology, and health care access.^9^, ^10^, ^11^, ^12^ Previous studies have consistently shown that Black/AA persons with MM have higher survival rates than their White counterparts when provided equitable care.^13^ This finding also holds true among participants in U.S.‐based clinical trials.^11^, ^14^ To address MM‐related disparities, ensuring equitable health care access and fostering trust is essential.^5^

The U.S. Public Health Service Untreated Syphilis Study at Tuskegee and other experiences of unethical research, both historical and contemporary, especially among Black/AA persons, have contributed to reduced health care system interactions and higher mortality rates, worsening racial health disparities.^15^, ^16^, ^17^, ^18^, ^19^, ^20^, ^21^, ^22^ The Tuskegee study, which aimed to evaluate the natural progression of syphilis among Black men in Tuskegee, Alabama, lasted from 1932 to 1972 and involved deceptive practices and denial of treatment, resulting in devastating consequences.^18^, ^19^, ^22^

Beyond the Tuskegee Study, there have been other historical events such as the creation of the “immortal” HeLa cells,^23^, ^24^ forced sterilization among Black/AA persons in the 19th and 20th centuries^25^, ^26^ and contemporary health care experiences related to the COVID‐19 pandemic^20^ that have further heightened medical mistrust among Black/AA persons. Despite the significance of these experiences, to our knowledge, prior studies have not thoroughly examined how these historical and contemporary experiences shape medical trust and mistrust among older adults with MM and their informal caregivers. Hence, we conducted a qualitative study that aimed to broadly understand the MM‐related experiences of Black/AA and White dyads. Considering existing knowledge of the disparities in MM‐care access, we also sought to understand upstream factors influencing access, such as trust or medical mistrust. The objective of this manuscript is to report on trust and medical mistrust within the health care context to more comprehensively understand these phenomena.

## 2 METHODS

### 2.1 Study design and setting

We used an equity‐based approach guided by the National Institute for Minority Health and Health Disparities Research Framework.^27^ With a thematic qualitative design,^28^ we interviewed dyads (patient‐caregiver) from the University of North Carolina at Chapel Hill's Lineberger Comprehensive Cancer Center (UNC‐CH‐ LCCC) via remote videoconferencing (Zoom Video Communications Inc). All interviews were conducted from November 2021 to April 2022. The study adhered to the Declaration of Helsinki and received approval from the UNC‐CH LCCC Scientific Review Committee and the UNC IRB (IRB Number: 21–0837). We followed the Consolidated Criteria for Reporting Qualitative Research (COREQ) guidelines.^29^

### 2.2 Participant selection

Our study methods have been previously described.^30^ We used purposive sampling^31^ to identify potential participants through electronic medical record screening, provider referrals, and distributing recruitment flyers (Data S1) to UNC‐CH‐affiliated patient and caregiver networks. Recruitment flyers were revised with input from UNC‐LCCC Patient Family and Advisory Council members. We utilized in‐person (UNC‐CH outpatient Hematology clinics) and remote (telephone) approaches for recruitment. Eligibility criteria included age 55 or older, MM diagnosis for at least 6 months, and receiving active treatment or previously received two lines of systemic anti‐myeloma therapies. All enrolled patients were required to have an adult caregiver willing to participate.

To ensure equitable representation, we implemented tailored recruitment strategies to enroll similar numbers of Black/AA and White participants with MM. This approach was crucial given the disproportionate incidence and poor survival rates among Black/AA individuals with MM and the persistent disparities in research enrollment.^11^, ^12^, ^32^, ^33^ Our recruitment efforts involved maintaining a racially diverse research team, practicing cultural humility/sensitivity, prioritizing in‐person recruitment, and assigning a single‐trained qualitative interviewer to conduct all dyadic interviews.^30^ The study included 21 dyads (42 participants) who completed a self‐report sociodemographic survey and a single joint semi‐structured interview.

### 2.3 Data collection

#### 2.3.1 Self‐reported sociodemographic survey

Each participant (patient and caregiver) completed a verbal self‐report sociodemographic survey administered by J.M. (co‐author and qualitative interviewer) via phone. The survey captured race, ethnicity, sex, marital status, insurance status, income, education level, employment status, and zip code. J.M. recorded responses using REDCap^34^, ^35^ hosted at UNC‐CH. Sociodemographics were collected for descriptive purposes and stratified analysis.^30^

#### 2.3.2 Semi‐structured interview guide development

S.J.G., P.M., and J.M. developed the interview guide (Data S1). S.J.G. is a Black‐Barbadian female clinician–scientist specializing in geriatric hematology and qualitative research. P.M. is a White male senior qualitative research methodologist with extensive experience, and J.M. is a Black/AA female and, at the time, a Public Health focused graduate student, newly trained in qualitative methods.^30^ The interview questions drew from relevant quantitative literature on MM in older adults^36^, ^37^, ^38^ and focused on exploring treatment experiences, the health impacts of MM, and factors influencing health care access, such as trust, transportation, mobility, and social support.^39^ Additionally, each dyad member was asked to share their perspectives on the Tuskegee Study^18^, ^19^ and its potential influence on trust or mistrust of the health care system.

#### 2.3.3 Semi‐structured interviews

S.J.G., J.M., and P.M. conducted mock interviews to refine the question sequence and structure.^30^ J.M., the single interviewer, conducted remote joint interviews with each dyad as guided by Arksey et al.^40^ and Eisokovits et al.^30^, ^41^ Before each interview, J.M. explained the study goals and had no prior relationship with the participants.^42^ Interviews were audio‐recorded, and video was encouraged for nonverbal communication capture (e.g., emotional displays, silences, hesitations, verbal disfluencies, changes in body positioning, posture, and eye contact avoidance). Joint interviews provided comprehensive context and real‐time interaction between dyad members.^42^ The interviewer carefully observed verbal and nonverbal cues, noted them in post‐interview memos, and discussed them during team meetings.

A professional service generated the transcripts, and J.M. wrote post‐interview reflective memos. Transcripts were assigned study IDs (CG, caregiver, PT, patient) with self‐identified race of the dyad (Black or White). S.J.G. and J.M. reviewed the transcripts for accuracy. Data collection continued until sufficient depth and complexity of concepts were observed (conceptual depth) guided by Nelson's five criteria (1. a wide range of evidence supported by the data, 2. intricate connections between themes and concepts, 3. researcher discernment of subtleties in concepts, 4. the resonance of findings with the existing literature, and 5. potential for external validation).^30^, ^43^ Both deductive and inductive topics were analyzed, and higher‐level themes were constructed.^30^, ^43^, ^44^

#### 2.3.4 Participant incentives

Each dyad member received a $50 e‐gift card within 72 h of completing the interview.

### 2.4 Data analysis

Descriptive statistics were used to summarize the survey data. The qualitative analysis team included the P.I., three research assistants (2 Black/AA females and 1 White female), and two medical students (both Black/AA females) trained in qualitative methods .^30^ Qualitative analysis followed the Sort and Sift, Think, and Shift approach^45^ using ATLAS.ti v.9 for project management. This approach involved identifying patterns, creating episode profiles, and discussing emerging themes in weekly team meetings. Dyadic approaches to data analysis were utilized based on methods by Arksey et al.^40^ and Eisikovits et al.^41^ for confirmability and dependability.^46^ Consistent emerging themes across dyads were examined to condense findings, as previously reported.^30^

### 2.5 Rigor

We described the steps taken to ensure study rigor in an earlier publication.^30^ Confirmability was achieved through rigorous methodological approaches to data collection and analysis,^40^, ^41^, ^45^ regular team meetings, and reflective memoing to address potential research bias.^46^ Dependability was ensured by using a single interviewer, pilot testing questions, and comparing and condensing themes across transcripts.^46^ Credibility was established through context‐rich quotation diagrams, a transdisciplinary team for diverse perspectives, and triangulation of memos, diagrams, and written reports to sufficiently understand participants' experiences.^46^

### 2.6 Methods: data sharing statement

For original data requests, please contact grantlabmm@gmail.com

## 3 RESULTS

**FIGURE 1:**
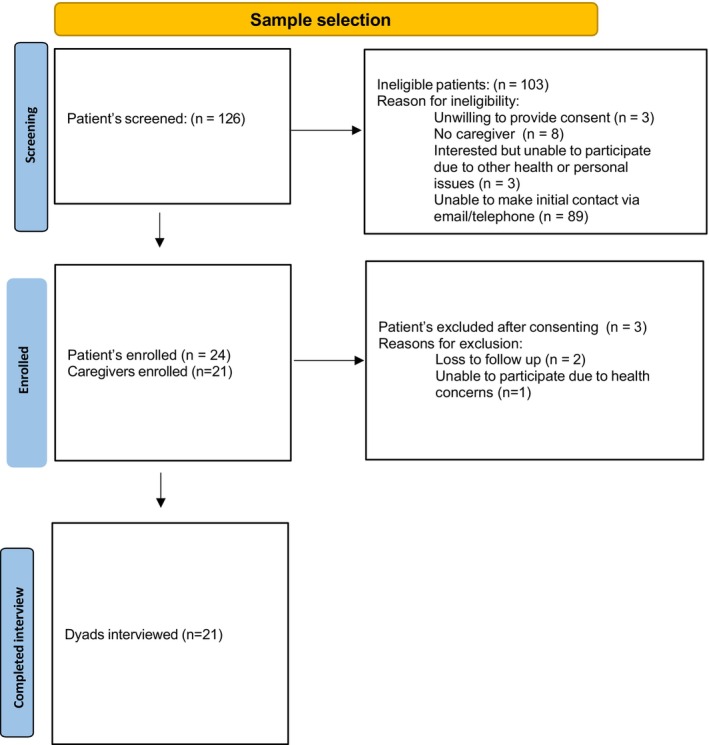
Demonstrates our sampling selection process. This figure indicates the population screened, consented, and enrolled and those who completed the semi‐structured interviews. This sample selection process also highlights participant reasons for ineligibility or exclusion from the study.

Out of 127 potential patient participants screened between November 2021 and April 2022, initial contact with 71% (*n* = 89) could not be made using a cold‐calling approach. Among those unreachable, 78% (*n* = 69) were Black/AA, as determined by the documented race in the electronic health record. Informed consent and survey completion were obtained from 24 patients and 21 caregivers. Ultimately, 21 dyads (*N* = 42) completed the interview. Figure 1 illustrates the sample selection process.

### 3.1 Sample sociodemographics

Participant sociodemographics are presented in Table 1. The interviewed dyads were racially concordant, with 11 Black/AA and 10 White pairs. Black/AA participants had a mean enrollment age of 70 (range: 60–80), while White patients had a mean enrollment age of 72 (57–90). Twenty‐three (55%) of all participants reported being assigned female sex at birth. Most caregivers (*n* = 18, 90%) were spouses or intimate partners, with 13 (62%) females. Both Black/AA and White caregivers had a mean enrollment age of 68. The mean number of years from MM diagnosis to enrollment for all patients was 5.5 years (standard deviation ±4).

**TABLE 1:** Sociodemographics of enrolled participants(30).

Participant characteristics	Black/AA dyads (*n* = 11) No./mean (%/SD)	White dyads (*n* = 10) No./mean (%/SD)	Total *N* = 42 participants No./mean (%/SD)
Patient–caregiver relationship			
Spouse/intimate partner	20 (91)	18 (90)	38 (90)
Adult child	2 (9)	2 (10)	4 (10)
Age			
Patient	70 (range: 60–80)	72 (range: 57–90)	71 (range: 57–90)
Caregiver	68 (range: 54–83)	68 (range: 37–88)	68 (range: 37–88)
Sex assigned at birth			
Male patient	5 (23)	6 (30)	11 (26)
Female patient	6 (27)	4 (20)	10 (24)
Male caregiver	5 (23)	3 (15)	8 (19)
Female caregiver	6 (27)	7 (35)	13 (31)
Ethnicity (self‐identified)			
Non‐Hispanic Latino/Latina	0 (0)	1 (5)	1 (2)
Marital status			
Married	20 (95)	18 (90)	38 (90)
Never married	0 (0)	2 (10)	2 (5)
Divorced	1 (5)	0 (0)	1 (2)
Widowed	1 (5)	0 (0)	1 (2)
Education			
High school	4 (18)	1 (5)	5 (12)
Some college/associates degree	7 (32)	4 (20)	11 (26)
Bachelor's degree	6 (27)	8 (40)	14 (33)
Master/doctoral degree	5 (23)	7 (35)	12 (29)
Median household income			
Below $50,000	6 (27)	4 (20)	10 (24)
$50,000–$99,999	11 (50)	6 (30)	17 (40)
Above $100,000	4 (18)	6 (30)	10 (24)
Prefer not to answer	1 (5)	4 (20)	5 (12)
Patient insurance status			
Medicare/medicaid	8 (73)	8 (80)	16 (76)
Private insurance	6 (55)	7 (70)	13 (62)
VA/Military	2 (18)	0 (0)	2 (10)
*Note: some have multiple insurance types			
Patient stem cell transplant history			
None	4 (36)	3 (30)	7 (33)
1–2 autologous	7 (64)	7 (70)	14 (67)
Patient current phase of treatment			
Maintenance (single drug)	1 (9)	5 (50)	6 (29)
Multidrug treatment regimen	7 (64)	5 (50)	12 (57)
Did not report	3 (27)	‐	3 (14)

*Note*: Demographics reported in this table have also been previously published in part in an earlier publication by Bates‐Fraser et al.^30^

Abbreviations: No, number; SD, standard deviation; VA, Veterans Affairs.

### 3.2 Qualitative data findings

Qualitative data findings revealed four major themes: (1) knowledge and its impact on trust, (2) experiencing heightened emotions and discomfort, (3) differing mental constructs of present‐day health care experiences, and (4) mitigating medical mistrust. Table 2 highlights themes according to participants' self‐reported racial identity, focusing on racial differences in line with the existing literature.^3^, ^16^, ^47^, ^48^, ^49^ The majority of participants showed a clear understanding of how knowledge and experiences regarding unethical research practices influence disparate health care experiences. Black/AA participants highlighted the lasting effects of events like the Tuskegee study on their beliefs and willingness to engage in research. Themes underscore the role of knowledge in building trust with health care providers and the health care system.

**TABLE 2:** Themes and example quotations from semi‐structured interviews with patient and caregiver dyads.

Themes	Example quotes	
Theme 1: Knowledge And Its Impact On Trust	“Tuskegee Experiment was awful. It was awful. And I think we've come a little bit too far to just trust.” **(Dyad 20 CG Black)**	“I don't think I've actually thought about that [Tuskegee study]. Let me just think. I'm thinking about this right now”…**(Dyad 11 PT White)**
	“Things are so closely watched now, and malpractice and all the stuff like that, it's not like it was back then. **(Dyad 14 PT Black)”**	“I have to think and consider [References the Tuskegee Study]. Now we're aware of all the, I call them atrocities of the past, and I just hope it's all in the past. It'll never happen again. I don't think it will, but I don't know. Now, things don't look too good looking down the pike.” **(Dyad 03 PT White)**
Theme 2: Experiencing Heightened Emotions And Discomfort	“A lot of our people suffered, suffered for what was done in the hospital…there was a lot of things that they practiced, I believe they practiced on you ‘cause they didn't have research and things like they have now’ **(Dyad 14 PT Black)”**	“Well, it could make me cry if I thought about [Tuskegee] because we're all people. To think that someone's gonna get treated differently because of the color of their skin is just no. It's just … I don't know, that's very hard for me (crying begins) **(Dyad 5 PT White).”**
Theme 3: Differing Mental Constructs Of Present‐Day Health care Experiences	“when the [references doctors and researchers] don't know—when they're trying to figure out what the medicine's gonna do or not do…you wanna trust that they take their oath seriously, for to do no harm. But sometimes everybody ain't had the right heart. For the research purposes, they sacrifice you for research purposes just to find out cause they have to find out what the medicine does” **(Dyad 20 CG Black)**	“I trust them [healthcare providers]. You have to trust them with all you got ‘cause I can't do this stuff [references medical care]” **(Dyad 17 PT White)**.
	“The [suggested MM therapy] was fairly new… and they wanted to do a clinical study, which would've been more like a guinea pig” **(Dyad 7 CG Black).**	“We feel like there are political agendas behind much of this COVID stuff. Many patients have not been given the treatment they need because they are older and very, low income and I believe there is a group that would say, get rid of the weak population and just do away with them, and let's get us a strong, young group of people that are left in this world that we can manipulate and control to bring about what we wanna bring about **(Dyad 6 PT White)**.”
	“So as far as the care, whether you are low‐income, middle‐income, or high‐income, I do believe when you're going to [NAME of ACADEMIC INSTITUTION] hospitals like that, these teaching hospitals that they, they do what they're supposed to do, makes no difference whether you're on Medicaid or whatever. I believe that. Now, I have seen, I have seen ‘cause I've been in the medical field, I've seen where private doctors like the area we come from are 1% Black and it's [TOWN NAME]. [I know of an office where the] doctor would take a Medicaid patient if another doctor referred, but for you to call and make an appointment and say, “Well, I have Medicaid,” no. He [the doctor] wouldn't see the patient: No, nope, nope, nope. It's interesting because they're not supposed to turn you down.” **(Dyad14 PT Black)**	“I don't think it does affect us and especially for me having been in the [NAME] system, I just, I'm aware of all of that. I mean, it's terrible that all of that happened, but it didn't happen to people we know, and being in the [NAME] profession, running experiments and things now for the last few years, there's so many safeguards built in what researchers can and cannot do, I don't see that kind of stuff happening again to the general public.” **(Dyad 12 CG White)**
	“Although she was a [community‐based] hematologist oncologist, she just treated patients. She didn't do any research or anything like that. She was just a doctor who treated multiple myeloma patients. And that didn't make me feel too comfortable, so I decided that I wanted to be treated at a hospital or facility where they did research, did clinical studies, they were on the cutting edge of what was happening as far as research and treating multiple myeloma.” **(Dyad2 PT Black)**	[In response to knowledge about the Tuskegee study] “Well, I don't know about the whole system, but everyone that I've had contact with, I think, has been very competent. They're just doing their job. I mean, if somebody makes a booboo, I mean, all of us make booboos… I mean, I think they're doing a great job. I—I just do.” **(Dyad 16 PT White)**
Theme 4: Mitigating Medical Mistrust	“… so we're well‐informed well before we go, even go to the doctors…so they can understand that…we kinda know what we're talking about. **(Dyad 19 CG Black).**”	“I have to trust them [DOCTORS] because I really don't trust anybody. I mean it's just the way the world is today.. you never know who's trying to get you or take advantage of you or you know and that's from everybody all the way up to in the medical field or up through the government. You can't trust anybody, you just gotta make your mind up, you just gotta trust in it and you know kind of go with your gut.” **(Dyad 17 PT White)**“Just know that they [DOCTORS] want what's best for you…just trust that God's gonna keep you pointed in the right direction and give you that feeling that this is what you need to do so let's do it.” **(Dyad 17 CG White)**
	“…there's a term that says that because of the lack of knowledge, we fail. Although during that time, so many African Americans were mistreated but, it was because there was a lack of knowledge. They didn't know what they were getting ready to go through; they didn't know what we agreed to. We attempted to get into studies when we first found out he had multiple myeloma, not because we knew that not because we was aware that a lot of African American men were having it, we had no clue. But we did know that this was something that couldn't be cured. And without the studies, there's a possibility there'll never be a cure. We know how important the studies are. But those that's involved in the study has to have some knowledge too. You can't just rely on what the doctors say; you gotta do your homework also.” **(Dyad 24 CG Black)**	“You've got to do your research, either on your own or through another medical person who has the resources to help you tap into the very best medical team that you can put together for yourself. You've got to be involved. You cannot put your head in the sand and just trust **(Dyad 6 PT White)**

Abbreviations: PT, patient; CG, caregiver.

### 3.3 Knowledge of research harmful to Black/AA persons and its impact on health beliefs (Theme 1)

Knowledge of research involving harm to Black/AA individuals, such as the Tuskegee study,^18^ forced sterilization practices,^25^, ^26^ and the development of the Henrietta Lacks immortal cell line,^23^, ^24^ differed between Black/AA and White participants. White participants sometimes needed more time to respond or were unfamiliar with these events. For example, “*I'm not familiar with that*. *So were there African Americans that were harmed*? (Dyad 6 PT White)”. In contrast, Black/AA participants shared personal anecdotes from their childhood that still influenced their health beliefs and resulted in medical mistrust. For example:

“*years back, the way they did treat Blacks, they did experiments on Blacks, and I don't like to say I heard a lot about this, but I was born and raised in [CITY NAME]. The things I heard, like when I was coming up as a kid about Tuskegee and all that stuff, I heard all of that… I said that's hard to believe, but it's true…* (Dyad 14 CG Black).”

This deeply rooted knowledge among Black/AA participants also influenced their present‐day beliefs about MM. One patient participant stated,

“w*ell, I sure hope it's not like the Tuskegee Institute–Experiment. Because if there's a cure for it [multiple myeloma], I wish they would give it to me—so that I can be done with this. In the back of our minds, we think about what happened down in Tuskegee when they had the cure all along—and didn't give it to them* (Dyad 22 PT Black).”

Finally, these beliefs were not limited to individuals with MM but reportedly remained deeply rooted within the broader Black/AA community. For example, “… *well some of the older people they don't like going to the hospital, don't like going to the doctors… especially in the South. They don't trust it [the health care system] so much*. (Dyad 14 PT Black).”

Our analysis highlighted racial disparities in knowledge and the impact of harmful medical research on Black/AA individuals, which could perpetuate a trust gap in U.S health care access between Black/AA and White persons and further exacerbate health disparities.

### 3.4 Experiencing heightened emotions and discomfort when discussing research harmful to Black/AA persons (Theme 2)

Racial differences were observed in participants' emotions and comfort levels when discussing the Tuskegee study and related events. Black/AA participants displayed directness and articulation, self‐initiating conversations on the topic. In contrast, White participants exhibited varying levels of discomfort, evidenced through nonverbal cues such as emotional displays, hesitations, changes in posture, and avoidance of eye contact. Similar communication patterns were observed within most dyads, except for two White dyads with noticeable tension between dyad members.

White participants' conscious awareness of events like the Tuskegee study led to some participants crying during the interview. One patient participant shared,

“*to pinpoint somebody because of their race or their religion or just the color of their skin. To pinpoint them and pull them out and abuse them or misuse them or withhold from them the quality medical treatment that they need whether they can afford it or not, whether they're of a specific ethnic group or specific race, to not make the very, very best available to them that they would for anybody else, that is beyond what my heart can hold* (*Dyad 6 PT White*).”

Some White participants expressed empathy toward Black/AA persons after learning about and reflecting on the Tuskegee study and similar events.

While our primary focus is to report the participants' experiences, we recognize the emotions experienced by the interviewer, J.M., during interviews and team meetings. J.M. acknowledged the discomfort in discussing the topic, particularly with White participants. In one interview, a White patient participant expressed empathy, saying, *sorry, I know it's hard for you too, but that's very hard for me when we see what has happened in the past* (Dyad 5 PT White).

Racial differences in emotional responses and comfort levels were observed during discussions of the Tuskegee study and similar events. Despite the pain these events may bring, especially for Black/AA persons, our findings emphasize the importance of educating others about this history and its ongoing impact on health care experiences, particularly for Black/AA persons.

### 3.5 Differing mental constructs of present‐day health care (Theme 3)

Black/AA participants' mistrust of the health care system significantly influenced their beliefs and experiences compared to White participants. A Black/AA caregiver stated, “*well, I believe it's still happening to Black patients… Black patients don't get the attention, don't get the care that our counterparts does*… (Dyad 22 CG Black).” In the context of MM, a caregiver shared their beliefs of systemic medical disregard of Black patients, for example,

“*our doctor has said that multiple myeloma mostly happens to Black people…which I don't want to believe… I'm thinking maybe that's why it is no cure, didn't study it… if it's just for Black people, so you don't care to study it* (Dyad 22 CG Black).”

Another noted, “we all know that things are not equal for us [Black persons], they're better as far as the eye can see, but they're not good (Dyad 20 CG Black).”

Trust in medical research was a frequent topic of discussion, particularly concerning drug‐related clinical trials. Black/AA participants often expressed fear and mistrust of these trials, leading to their nonparticipation. However, some Black/AA participants exhibited absolutist thinking, forming a mental model of their perceptions of trials. For instance,

“…*when they start talking about any medicines… and they like to do all these different trials… NO! When it comes to putting stuff in the body? I can't do it. Even though I know some people get the actual medicine and some get the placebo, that's fine; that's great for those people. But I, I don't like for us to do that*. (Dyad 20 CG Black).”

Medical research, including clinical trials, was perceived negatively by participants, particularly among Black/AA persons who expressed feelings of being used as “guinea pigs” or as experiments. Additionally, some participants mentioned COVID‐19 vaccine studies as contributing to research mistrust among Black/AA persons. For example,

“*I don't want to be a guinea pig. When you do the clinical trials, I want to make sure it's like it was or like it is rather with the coronavirus vaccine. How many people were tested prior to you wanting to give it to the masses? So I'm not afraid of science if used properly, but if it's gonna be misused, you will not misuse it on me if I can help it* (Dyad 18 PT Black).”

A few White participants highlighted the differential treatment of COVID‐19 based on sociodemographics, which shaped their beliefs about the underlying factors driving observed COVID‐19‐related health care disparities.

White participants demonstrated higher levels of trust in the health care system compared to Black/AA persons. Many White participants also recognized the privilege associated with their race and how it influenced their beliefs and trust in the health care system. One patient participant mentioned and acknowledged her White privilege:

“*I trust the medical system, and I know that's White privilege. I believe the Tuskegee airmen's experiences weren't general throughout the population. I think they were targeted because they were considered more expendable. I know that's terrible, but it didn't affect my trust in the medical system*. *Maybe it should have…* (*Dyad 10 PT White*).”

This quote demonstrates the self‐reflection that some participants experienced during interviews.

While some White participants showed empathy toward the experiences of Black/AA persons, they also acknowledged the differential impact of their knowledge of such events. One White patient participant recognized the potential difference in trust between themselves and a Black individual in the context of a clinical trial with a new medication. For example,

“*I think there's a lot of trust I might have as a White person going in for a clinical trial with a new medication that I do not know that someone with comparable living experiences…who is Black would feel* (Dyad 11 PT White).”

The patient's caregiver shared a similar perspective, speculating that if they were Black, they would likely be more distrustful or cautious due to the historical context, “*I can only really guess that if I were Black, I'd probably be very distrustful or leery more. I don't have that history that might scare me*… (Dyad 11 CG White).”

Some participants, particularly White individuals, struggled to empathize with the experiences of Black/AA persons. One participant did not perceive bias against Blacks with myeloma and could not imagine differential treatment, as noted, “*I just don't see any bias against Blacks with myeloma, and I can't imagine any myeloma specialist treating patients differently* (*Dyad 11 CG White*).”. Another participant acknowledged the challenges Black/AA persons faced but felt it didn't significantly impact their own life,

“*it doesn't affect my life; we're aware of it. Sucks what has happened in the past and which still goes on … but it doesn't change my life other than I'm aware of it … we have sympathy for it or empathy* (Dyad 12 PT White).”

Our findings showed that Black/AA participants were more mistrustful of the health care system than White participants. While White participants expressed empathy, their overall trust in the health care system remained relatively unaffected.

### 3.6 Mitigating medical mistrust (Theme 4)

Acknowledging the history of medical mistrust, Black/AA participants especially emphasized the importance of educating themselves to gain more knowledge about the disease. This desire for self‐learning also encompassed the idea of patients and caregivers “*doing their own research*,” for example,

“*as a result of having knowledge of [Tuskegee] … we don't just depend on the doctors to tell us what to do or to advise us the direction that we should go as far as treatment. We do our research*. (Dyad 2 PT Black).”

This knowledge gained from self‐learning was seen as empowering and garnering respect from health care providers, allowing participants to express themselves confidently; for example, “*it does help when you have some knowledge about medication or the medical field; you can explain yourself in your terms. I think it's brought some respect* (Dyad 14 PT Black).”

The ability of patients and caregivers to self‐learn about MM influenced decision‐making on offered MM therapies. One participant emphasized the importance of reading and not unquestioningly accepting information provided by health care professionals, “*that's why I read…you don't tell me something and think I just accept it because you said it because I know what my situation is medically, and I need to know what's out there* (Dyad 18 PT Black).” However, participants also acknowledged the need to balance the information they received from different sources as part of self‐learning and their proactive approach to care.

“*I'm gonna do the treatment that I feel is best for me based on my research and what the doctor tells me, so it would behoove the doctor, don't leave out any vital information that I might need to make an intelligent decision. I look at it as the doctor and us, me and my wife, we partner together for my treatment, the treatment plan, and the strategy. It's a partnership, and all of us are working together…. I understand he [the doctor] has the credentials and the experience in treating the disease cause I'm not a doctor*.” (Dyad 2 PT Black)

Participants viewed this approach as crucial to ensuring they had access to the best possible care.

Trust emerged as a central theme for Black/AA and White participants in their relationships with health care providers. One participant emphasized the time it takes to build trust and how easily it can be lost, “*trust takes years to gain and a second to lose* (Dyad 5 CG White).” A Black caregiver highlighted the importance of trusting the doctors: “*I think you have to have a peace of mind about who's caring for you too…you have to trust your doctor and, hoping that they have your best interests at heart* (Dyad 23 CG Black).” While trust in providers was acknowledged, participants also emphasized the need for self‐efficacy, “*I also feel, for us, that you have to be proactive with your own care, making sure that people are doing what they're supposed to be doing* (Dyad 16 CG White).” Trust plays a significant role in shaping patients' and caregivers' beliefs and perceptions about health care.

## 4 DISCUSSION

This qualitative study provides contextual insights into the persistent challenges of medical mistrust, especially within the Black/AA community, and the implications for patients and caregivers accessing and receiving MM‐related care and their broader interactions with the health care system. Our findings highlight racial disparities in knowledge acquisition timing, emotional impact, and the overarching impact of previous unethical research practices particularly harmful to Black/AA persons on present‐day trust in health care. Cultivating trusting relationships among patients, caregivers, and health care providers emerged as a pivotal strategy identified by participants to bridge the trust gap and enhance health care experiences, especially within the Black/AA community.

Like previous studies,^3^, ^22^, ^50^ our study focused on trust, using the Tuskegee study as a symbolic historical event that helped introduce the topic. However, our semi‐structured interviews allowed participants to discuss other historical and contemporary events contributing to medical mistrust. Consistent with Alsan and Wanamaker's findings, Black/AA participants frequently expressed mistrust, shaping their beliefs and health care system interactions.^22^ These beliefs also influenced their perceptions of MM, including its disproportionate impact on Black/AA individuals and its status as a largely incurable cancer.

Our data revealed contrasting discourse communities formed among Black/AA and White participants based on historical events. Black/AA participants demonstrated a discourse community shaped by the enduring impact of events like the Tuskegee study, fostering perceptions of ongoing racial discrimination in health care. In contrast, White participants exhibited a discourse community influenced by historical medical advantage. This perspective led some White study participants to believe that there was no current evidence of discrimination against Black/AA individuals with MM or in their interactions with the health care system. These findings align with a 2020 survey where a higher percentage of Black respondents (70%) compared to White respondents (41%) agreed that the health care system mistreats people based on race or ethnicity.^51^ Moreover, fewer Black/AA participants in that study expressed trust in their providers compared to White participants (59% vs. 78%).^51^

In our study, Black/AA participants acknowledged the transgenerational emotional burden of the Tuskegee study, which shaped their perspectives on trust in health care. J.M., the interviewer, also felt this emotional burden during interviews. White participants, mostly unfamiliar with the Tuskegee study, showed varied emotional responses upon learning or recalling these events. While some expressed empathy, their trust in health care remained largely unaffected. The transgenerational knowledge among Black/AA participants not only affected individuals but was also seen to influence the overall trust of the community in the health care system. This finding is crucial as medical mistrust is linked to lower health care utilization,^52^, ^53^ increased symptom burden, and advanced stage at initial cancer diagnosis,^54^, ^55^ poor adherence to recommended medical therapies,^56^ and limited engagement in medical research.^3^ These outcomes are particularly relevant to MM, as Black/AA persons fear worse regarding access, timely care, quality treatment options, and survival.^9^, ^13^, ^57^, ^58^, ^59^, ^60^, ^61^

While both Black/AA and White participants stressed the significance of self‐educating themselves about MM, Black/AA participants were more open in their discussions about it and displayed a predominant inclination toward the idea of “*doing your own research*” to cultivate trust in recommended medical care. Both Black/AA and White dyads highlighted the significance of relationships with providers in building trust. These findings support the need for consistent, transparent, and empathic communication between patients and providers to shape health care experiences.^62^, ^63^, ^64^ Building trust takes time. One participant stated, “*trust takes years to gain and a second to lose*.” This perspective is crucial for future interventions designed to address medical mistrust in health care.

### 4.1 Strengths and limitations

Strengths of our study include equal enrollment of Black/AA and White participants, allowing diverse perspectives. Focusing on participants in NC provided a deeper understanding of a specific region. Joint interviews revealed roles within dyads and created space and support, allowing each dyad member might reinforce and build upon each other's accounts, generating richer and more nuanced data.^30^, ^40^, ^41^

However, limitations emerged. Initial cold‐calling led to a 78% nonresponse rate, highlighting recruitment challenges tied to the lack of an established relationship or prior rapport between the study team and potential participants. Many also avoided answering our calls, which may have been from numbers unfamiliar to them, an observation aligned with a 2020 Pew Research survey indicating that 8 out of 10 Americans avoided such calls from unrecognized numbers.^65^ Despite this, our in‐person recruitment efforts and methodological rigor, including a conceptual depth criterion for sample size estimates,^43^ likely mitigated the impact of the high nonresponse rate.

Another limitation was that we conducted all interviews via videoconferencing, which could hinder participation due to technology disparities influenced by race/ethnicity and socioeconomic status.^66^ While our study findings can not represent the entire U.S. population, our findings could resonate more broadly due to entrenched mistrust among Black/AA persons in the U.S. Lastly, we focused on older adults with MM, specifically those who had received ≥2 prior therapies or ≥6 months post‐diagnosis, along with adult caregivers. The translation of our findings to other patient‐caregiver groups, like those with newly diagnosed MM, is limited.

## 5 CONCLUSION

Our study findings offer valuable contextual insights into the enduring challenges related to trust and mistrust, especially within the AA/Black community, and how these challenges reverberate within health care. Several participants highlighted the pivotal role of patient‐oncologist relationships, and the ability to form personal connections with their health care team. These relationships were deemed crucial for building trust and addressing concerns held by patients and caregivers, spanning aspects like myeloma treatments, clinical trials, and the health care system. Another fundamental issue discussed among dyads was the need for improved communication between providers and patient‐caregiver pairs.

We advocate that future studies harness our findings as a guiding framework when crafting interventions to cultivate trust between patients, family members, and the health care system. Such future efforts could leverage successful models like the “3c model” (3rd Conversation).^67^ The “3c model” creates a virtual space where patients and clinician groups and clinician‐health care system administrator groups meet to mutually comprehend health care experiences from all perspectives. This model seeks to foster relationships, encourage empathetic and patient‐centered communication, and aid patients and families in navigating the health care system more adeptly. In the 3c model, conversations with health care system administrators offer the chance to develop enduring solutions that can drive changes at the systems level.

This strategic approach has the potential to address medical mistrust at the population level in the future. It moves beyond the individual and interpersonal changes achievable through community‐based navigation and education approaches^68^ by aiming for broader improvements in the health care system. As this model expands, its influence could extend to encompass societal transformations.

Finally, we also underline the significance of policy‐level interventions, exemplified by initiatives like the Center for Disease Control's Racial and Ethnic Approaches to Community Health^69^ and the National Cancer Institute's Center to Reduce Cancer Health Disparities.^70^ These efforts remain crucial in advancing health equity and alleviating the existing burden of racial health disparities.

## AUTHOR CONTRIBUTIONS

**Shakira J. Grant:** Conceptualization (lead); data curation (lead); formal analysis (lead); funding acquisition (lead); investigation (lead); methodology (lead); project administration (lead); resources (lead); software (lead); supervision (lead); validation (lead); visualization (lead); writing – original draft (lead); writing – review and editing (lead). **Jiona A. Mills:** Conceptualization (supporting); data curation (supporting); formal analysis (supporting); investigation (equal); project administration (supporting); software (supporting); validation (supporting); visualization (lead); writing – original draft (equal); writing – review and editing (equal). **Joseph Telfair:** Methodology (supporting); project administration (supporting); writing – original draft (equal); writing – review and editing (equal). **Gabriell Erisnor:** Conceptualization (supporting); data curation (supporting); formal analysis (supporting); methodology (supporting); software (supporting); writing – original draft (equal); writing – review and editing (equal). **Tanya M. Wildes:** Writing – original draft (equal); writing – review and editing (equal). **Lauren C. Bates‐Fraser:** Conceptualization (supporting); data curation (supporting); formal analysis (supporting); writing – original draft (equal); writing – review and editing (equal). **Andrew F. Olshan:** Writing – original draft (equal); writing – review and editing (equal). **Erin E. Kent:** Writing – original draft (equal); writing – review and editing (equal). **Hyman B. Muss:** Writing – original draft (equal); writing – review and editing (equal). **Paul Mihas:** Conceptualization (equal); formal analysis (equal); methodology (equal); project administration (equal); software (equal); supervision (equal); writing – original draft (equal); writing – review and editing (equal).

## FUNDING INFORMATION

LBF is supported by the National Cancer Institute's National Research Service Award, sponsored by the Lineberger Comprehensive Cancer Center at the University of North Carolina Grant No. (T32 CA116339) and The University of North Carolina Lineberger Comprehensive Cancer CenterUniversity Cancer Research Fund. The project was supported by the National Center for Advancing Translational Sciences (NCATS), National Institutes of Health Grant No. UL1TR002489. GE is supported by the National Institute on Aging 2022 Medical Students in Aging Research (MSTAR) program grant at the University of North Carolina‐ Chapel Hill (UNC‐CH) Grant No. (NIA T35AG038047). SJG is supported by National Cancer Institute Grant No. (5‐K12‐CA120780‐13; PI: William Kim) and National Institute on Aging Grant No.(1 R03 AG074030‐01; PI: Shakira Grant). The funder had no role in the study's design, the collection, analysis, and interpretation of the data, the writing of the manuscript, or the decision to submit the manuscript for publication.

## CONFLICT OF INTEREST STATEMENT

The authors declare no relevant conflicts of interest.

## DISCLAIMER

The content is solely the responsibility of the authors and does not necessarily represent the official views of the National Institutes of Health.

## ETHICS APPROVAL STATEMENT

This study was conducted per the Declaration of Helsinki and approved by the UNC‐LCCC Scientific Review Committee and the UNC Institutional Review Board (IRB Number: 21‐0837).

## PATIENT CONSENT STATEMENT

Informed consent was obtained from all patients and caregivers participating in this study.

## CLINICAL TRIAL REGISTRATION

This study did not meet the criteria defined by the National Institutes of Health (NIH) for a clinical trial, and, as a result, it was not registered on ClinicalTrials.gov.

## Supporting information


Data S1:

## Data Availability

For original data, please contact grantlabmm@gmail.com
