# Supplementary material for: “They don't care to study it”: Trust, race, and health care experiences among patient‐caregiver dyads with multiple myeloma
Source: Cancer Med. 2024 May 21;13(10):e7297. doi: 10.1002/cam4.7297 (PMC11106687; doi:10.1002/cam4.7297)
Supplement: Supplementary file 1 — Data S1: [file CAM4-13-e7297-s001.docx]

**Supplemental Materials**

**Supplemental Materials include the following items**

**Supplemental File A** is the recruitment flyer used to disseminate information and recruit potential participants for the study.

**Supplemental File B** is the full semi-structured interview guide used in this study

**
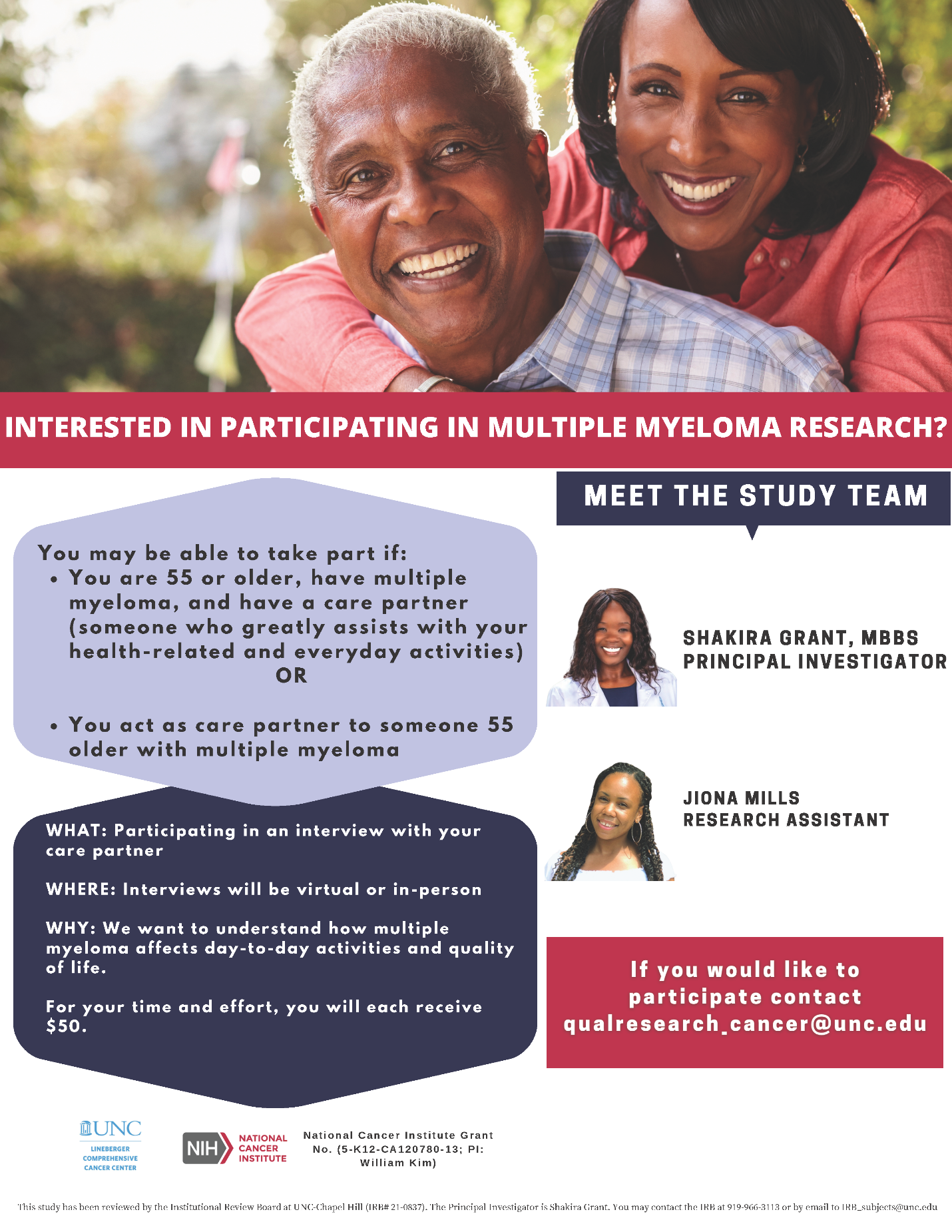
Supplemental File A**

**Supplemental File B**

**Interview Guide.**

**Section I. Introduction**

1. To begin, can you tell me your story of how you came to be diagnosed with multiple myeloma? (patient)
2. if not addressed: Can you tell me how long you have been living with multiple myeloma?

2.   Thinking back to when your [INSERT RELATIONSHIP TO PATIENT HERE] was first diagnosed with multiple myeloma, can you tell me what that experience was like for you? (caregiver)

1. Can you tell me what your life was like before being diagnosed with multiple myeloma (patient and caregiver)
2. What are some of the things you enjoyed doing before your myeloma diagnosis?
3. Are there specific things you enjoyed doing together with your [INSERT RELATIONSHIP HERE to the patient] before your myeloma diagnosis?

**Section II. Myeloma-focus**

1. What has your life been like since being diagnosed (and while receiving multiple myeloma therapies) (patient and caregiver)
2. What, if anything, has changed in your daily life? (patient and caregiver)
3. How would you describe multiple myeloma in your own words? What does it do to you?
4. What are the primary problems that multiple myeloma has caused for you?
5. What do you fear most about multiple myeloma?

1. Since you were diagnosed with multiple myeloma and began receiving treatments, have you noticed any changes in the kinds of things you can and cannot do?
2. What gets in the way of doing certain activities?
3. What things do you wish were easier to do?
4. What, if anything, helps make it possible to do things that are now more difficult?
5. If not already addressed above then probe around physical limitations: for example, is it more difficult getting to your doctor's appointments, getting groceries or medications, or walking, can ask about their memory for example: Do you forget things more easily than before?

1. How, if at all, has multiple myeloma and the different types of **treatments** you've received affected your relationship with others? Ask specifically about the patient-caregiver relationship (patient perspective and the care-partner perspective)
2. How, if at all, has multiple myeloma and the different types of treatments you've received affected your quality of life?

**Section III. Treatment-focus**

1. Can you tell me about the kind of treatments you have received for your myeloma?
2. What have those been like for you?
3. Probe- Which of the treatments have been the most challenging for you?
4. Probe- Is the treatment what you would've expected?

1. Thinking about past events where Black patients may have been deliberately harmed during research experiments  such as the Tuskegee experiment.
2. What do these events mean to you?
3. How do these events affect you and how you interact with the medical system (example: the types of treatments you would be willing to receive), medical research, or your health care providers?
4. How do people in the Black community that you have been exposed to react to the medical system, medical research, or health care providers?
5. To what degree to do trust the medical system to treat you and your illness? (Ask of Black and White patients)

1. When thinking about your myeloma treatments, what are the most important results you would hope to receive from them?

1. When discussing treatment options with your doctor, how involved are you and your caregiver in making decisions? Tell me more about what that conversation was like.
2. Can you think of a challenging conversation you had with your provider when discussing the next line of multiple myeloma treatment. Can you tell me what that conversation was like for you?
3. Did you mention your concerns about the treatments with them?
4. How did it feel when you brought up your concerns?
5. Did you feel that your doctor understood your concerns?
6. Is there something you wanted to talk about that you didn't bring up?
7. What are some of the things that you find helpful to know when discussing a new line of treatment for your multiple myeloma?

1. Can you tell me about any challenges you may have encountered getting to the cancer center for your myeloma treatments?
2. Probe around social barriers: transportation, for example can you think about a time when you had no way of getting to your doctor's appointment
3. Probe around the length of time spent traveling to and from appointments with the cancer doctor. Can you think of anything that concerns you (patient or caregiver) about the travel required for your cancer therapies?

1. What do you fear most about the future regarding your multiple myeloma treatments?

**Section IV. Future**

1. If you knew then (after your diagnosis) what you know now, what would you change about the care you have received for your myeloma?

1. What advice, if any, would you give someone who has just been diagnosed with myeloma? (Both patient and caregiver should respond to this.)

1. What advice would you give your providers about treating someone with multiple myeloma?

**Section V: Closing**

1. Is there something we have not covered that you would like to share about your experience with myeloma?
